# Sleep related movement disorders in the elderly: a review of recent literature

**DOI:** 10.3389/frsle.2024.1356644

**Published:** 2024-02-27

**Authors:** Marc Comair, Sandy Awad, Pritha Ghosh

**Affiliations:** Department of Neurology and Rehabilitative Medicine, School of Medicine and Health Sciences, The George Washington University, Washington, DC, United States

**Keywords:** sleep-related movement disorders, restless leg syndrome, periodic limb movement disorder, nocturnal cramps, bruxism

## Abstract

Sleep-related movement disorders (SRMD) are common, often troublesome, conditions in the elderly. Although these disorders can impact people of all ages, there are different considerations when diagnosing and managing SRMD in the elderly. In this review, we discuss SRMD in the elderly, focusing on recent developments in the areas of restless leg syndrome, periodic limb movement disorder, nocturnal muscle cramps, and sleep-related bruxism. In addition to reviewing these entities more generally, we highlight key considerations in addressing these in conditions in older adults.

## 1 Introduction

Sleep is increasingly recognized as a key factor in promoting healthy aging (Miner and Kryger, 2017; Gulia and Kumar, 2018). Although some age-related changes in sleep architecture are part of normal physiology, disorders of sleep, such as chronic insomnia, sleep-related breathing disorders, parasomnias, and sleep-related movement disorders are common in the elderly, affecting up to 40%−70% of older adults aged 65 and older (Miner and Kryger, 2017; Gulia and Kumar, 2018). Sleep-related movement disorders (SRMD) are one category of sleep disorders that can occur in the geriatric population which we will focus on in this review. The International Classification of Sleep Disorders (ICSD) defines SMRD as being “relatively simple, usually stereotyped movements that disturb sleep and its onset” (American Academy of Sleep Medicine, 2014). Although SRMD can encompass several different disorders, in this review, we will discuss common SRMD in the elderly, focusing on developments in these fields over the last 5 years. Specifically, we will discuss restless leg syndrome (RLS), periodic limb movement disorder (PLMD), nocturnal muscle cramps (NMC) and sleep-related bruxism (SB), highlighting recent updates that can directly impact clinical management in this age group.

## 2 Methods

The purpose of this paper is to review common sleep-related movement disorders in the elderly and discuss recent research related to SRMD management over the last 5 years. The MEDLINE database was used to identify papers published in English from January 2018 to November 2023 in adults. The search terms included: restless leg syndrome AND management; restless leg syndrome AND treatment; restless leg syndrome AND elderly; periodic limb movement AND management; periodic limb movement AND treatment; periodic limb movement AND elderly; nocturnal cramps AND management; nocturnal cramps AND treatment; nocturnal cramps AND elderly; bruxism AND management; bruxism AND treatment; bruxism AND elderly. As the definition of “elderly” varied across studies, we broadened our search to review papers that involved individuals age ≥55 years, except in the literature on bruxism, where nearly all studies have been done in younger individuals. Results appearing in more than one search or involving populations outside of the general older adult population were removed, aside from the literature on bruxism, where data in the elderly was very limited. Randomized controlled trials, cohort studies, case-control studies, observational studies, clinical trials, and meta-analyses were included, with attention to articles involving the care of older adults. The articles were independently reviewed by MC, SA, and PG. Initial searches yielded 421 results, and after filtering by the inclusion criteria, removing duplicates and reviewing full texts, 54 articles pertaining to recent updates in SRMD management were included in this review.

## 3 Results

### 3.1 Restless leg syndrome

Restless legs syndrome (RLS), historically known as Willis-Ekbom disease, is a common, chronic sensorimotor disorder characterized by an uncomfortable urge to move during rest. Patients present with symptoms that vary widely in frequency and severity (Gossard et al., 2021; Silber et al., 2021). The prevalence of RLS in the general population ranges from 1 to 20%, with a higher prevalence in women (Berger et al., 2004; Manconi et al., 2004, 2012; Allen et al., 2005; Tison et al., 2005; Gustavo Persi et al., 2009). RLS may be more common in European and North American populations (Ohayon et al., 2012) and there is an increased risk of RLS in pregnant or multiparous women (Berger et al., 2004; Manconi et al., 2004, 2012). Several, but not all, studies also suggest an increasing prevalence of RLS with age, affecting up to 18%−35% of people over the age of 40, with several reports of symptoms peaking around age 65 (Allen et al., 2005; Tison et al., 2005; Ohayon et al., 2012; Praharaj et al., 2018; Gossard et al., 2021).

The diagnosis of RLS is based on clinical symptoms that include uncomfortable sensations primarily in the legs that produce an urge to move the legs in order to seek relief. The symptoms tend to occur in the evening or at night and are triggered by prolonged rest or inactivity (Allen et al., 2014). The updated clinical diagnostic criteria from the International Restless Legs Study Group (IRLSSG) highlight the need to exclude mimics, such as nocturnal leg cramps, peripheral neuropathy, fibromyalgia, or motor akathisia. In addition, the IRLSSG outlines valuable supportive features in diagnosing RLS, such as an initial reduction in symptoms following dopaminergic treatment, first-degree relatives with RLS, a lack of profound daytime sleepiness, and periodic leg movements either during sleep (PLMS) or wakefulness (PLMW) (Allen et al., 2014). RLS can occur as a primary disorder, or secondary to other factors, such as iron deficiency, pregnancy, kidney disease, Parkinson's disease, or medications such as antidopaminergic medications and serotonergic or noradrenergic reuptake inhibitors (Gossard et al., 2021). Exploring and addressing these potential factors is a critical step in the diagnosis and management of RLS.

The underlying etiology and pathophysiology of RLS is complex and imprecisely understood. While multiple genetic risk loci have been identified that can contribute to the pathogenesis of RLS, there are a variety of modifiable risk factors associated with RLS, including obesity, alcohol consumption, smoking, and inactivity (Winkelmann et al., 2000; Didriksen et al., 2020). Several studies suggests that brain iron deficiency, characterized by low ferritin and high transferrin levels in the cerebrospinal fluid, is implicated in the pathogenesis of RLS (Gossard et al., 2021). This observation holds true even in the absence of peripheral iron deficiency, suggesting that brain iron acquisition, mobilization, or storage may be involved. Brain iron plays a key role in the synaptic reuptake of dopamine and as a co-factor for tyrosine hydroxylase, the rate-limiting enzyme in dopamine synthesis (Hare et al., 2013; Silber et al., 2021). Impairment in these processes is linked to reduced D2 dopamine receptor density, which is thought to underlie the key pathophysiological mechanism of RLS, explaining why dopaminergic medications can be effective treatments for RLS (Hare et al., 2013; Didriksen et al., 2020).

For patients with intermittent or mild RLS, defined as RLS that is non-burdensome or occurs less than twice weekly, a non-pharmacologic approach is often helpful to decrease symptom severity. Patient education on proper sleep hygiene is important, with a regular sleep-wake cycle and adequate sleep time being factors in controlling RLS symptoms (Aukerman et al., 2006). Exercise can also be helpful. Lower body resistance and aerobic exercise may be particularly effective in alleviating RLS symptoms, though the mechanism of the effect is poorly understood (Aukerman et al., 2006). Other measures that may be effective include massages, stretching, walking, light therapy, and nutraceuticals. While evidence is limited, these practices tend to be safe and tolerable for most patients (Aukerman et al., 2006; Bega and Malkani, 2016).

Chronic persistent RLS is defined as frequent and burdensome RLS symptoms occurring at least twice weekly. The management of chronic persistent RLS is focused on symptom alleviation and improving quality of life through addressing secondary causes of RLS, lifestyle modifications, non-pharmacological interventions, and pharmacotherapy (Garcia-Borreguero et al., 2016; Kolla et al., 2018; Elrassas et al., 2021). In patients with evidence of iron deficiency and serum ferritin <75 μ/L, supplementation with ferrous sulfate 325 mg with vitamin C 100 mg once or twice daily may improve RLS symptoms (Garcia-Borreguero et al., 2016; Allen et al., 2018; Silber et al., 2021). In the setting of moderate to severe chronic persistent or refractory RLS, impaired absorption, or intolerance to oral iron, IV ferric carboxymaltose is recommended as a first-line iron therapy for patients with serum ferritin levels <100 μ/L (Allen et al., 2018).

Dopaminergic drugs, such as levodopa, or non-ergot dopamine agonists, such as pramipexole, ropinirole, and rotigitine, have long been recognized as effective pharmacotherapy for chronic or refractory RLS (Garcia-Borreguero et al., 2016; Winkelman et al., 2016; Allen et al., 2018). However, these drugs are also known to cause augmentation of RLS symptoms over time (Garcia-Borreguero et al., 2016; Winkelman et al., 2016; Allen et al., 2018; Wanner et al., 2019). In addition, they can have other adverse effects including sedation, edema, and impulse control disorders (Gossard et al., 2021). Therefore, the 2016 international consensus guidelines recommended a shift to consider alpha-2-delta ligand medications, such as gabapentin, gabapentin enacarbil, or pregabalin, as first-line therapy to mitigate potential augmentation (Garcia-Borreguero et al., 2016). Alpha-2-ligand medications bind the alpha-2-ligand on calcium channels, which in turn, regulates the release of pre-synaptic neurotransmitters. While these medications can cause drowsiness, dizziness, leg edema, or weight gain and require modified dosing in those with renal impairment, they are generally well tolerated and have demonstrated efficacy, particularly in mild RLS (Gossard et al., 2021). In using dopaminergics, the international guidelines recommend starting with the lowest effective dose and to consider a longer acting dopaminergics or divided daily dosing if mild augmentation develops (Garcia-Borreguero et al., 2016). Low-dose, prolonged-released opioids may be considered in severe cases of augmentation (Garcia-Borreguero et al., 2016; Winkelman et al., 2016; Allen et al., 2018; Wanner et al., 2019). Finally, neurostimulation over the common peroneal nerve and transcutaneous spinal cord direct current stimulation have recently shown beneficial results in RLS patients, but further investigation is warranted (Wang et al., 2020; Buchfuhrer et al., 2021; Cho et al., 2022; Roy et al., 2023).

### 3.2 Recent updates in managing older adults with RLS

As RLS is common in the geriatric population, special attention to the implications or issues associated with RLS in this population is necessary (Table 1). For example, as older patients may have more medical co-morbidities or polypharmacy, it is critical to consider potential secondary causes of RLS by assessing for iron deficiency, renal disease and reviewing their medications (Kolla et al., 2018; Earley et al., 2021; Elrassas et al., 2021). The increased risk of nighttime falls due to nighttime ambulatory episodes is another important consideration (Kuzniar and Silber, 2007; Richards et al., 2021). Addressing balance problems and encouraging safety precautions such as properly lighting when ambulating at night is recommended.

**Table 1:** Considerations for managing restless leg syndrome and periodic limb movement disorder in the elderly.

**Restless leg syndrome**	
General considerations	• Review iron studies and metabolic panel. Treat iron deficiency and address renal concerns. • Review potential causative medications (dopamine blockers, SSRI, SNRI) • Consider fall safety precautions for elderly individuals ambulating at night • Carefully review potential side effects of pharmacotherapy in this age group based on co-morbidities • In those with dementia, consider RLS in the differential diagnosis of nighttime agitation • In those with dementia, avoid dopamine agonists due to potential neuropsychiatric side effects
Diagnostic considerations	• Assess for mimickers and comorbidities for RLS (Gossard et al., 2021) common in the elderly: ◦ Peripheral neuropathy ◦ Parkinson's disease ◦ Renal Insufficiency ◦ Cardiovascular disease ◦ Cognitive decline • Consider common medications that can be associated with RLS: ◦ Neuroleptics ◦ SSRI/SNRI ◦ Anti-dopaminergic antiemetics ◦ Proton-pump inhibitors ◦ Histamine H2-receptor antagonists • Check serum basic metabolic panel and iron studies
Recommendations for treating mild RLS (< twice/week)	• Education on sleep hygiene • Exercise (stretching, yoga, lower-body resistance, aerobic) • Massage
Recommendations for treating chronic RLS (>twice/week)	• Counsel on association of cardiovascular disease in untreated RLS • Start with non-pharmacological therapies (Sleep hygiene, exercise, massage) • For serum ferritin < 75 μ/L or transferring saturation < 20%−25% Gossard et al., 2021, start oral iron replacement [ferrous sulfate 325 mg (65 mg elemental iron) with Vitamin C 100 mg] or intravenous ferric carboxymaltose 1,000 mg Allen et al., 2018 • Alpha-2-ligands are first -line pharmacotherapy for RLS and may be effective for moderate severity RLS Garcia-Borreguero et al., 2016 • Dopamine agonists are effective for moderate to severe RLS, but can cause augmentation, impulse control disorder, sedation and edema. Use with caution and with lowest effective dose Garcia-Borreguero et al., 2016
**Periodic limb movement disorder**	
General considerations	• Diagnosed on the basis of polysomnography (>15 PLMS/hour) • Counsel on association of cardiovascular disease and PLMD • Assess for potential co-morbid cognitive decline • If PLMS is occurring with RLS, consider management options related to RLS

Reinforcing lifestyle modifications, including optimizing sleep hygiene, and physical exercise, remains a cornerstone of treating RLS in older adults (Table 1). A recent study has been done looking at the benefits of yoga in patients with a mean age of 50 years showed promising results, but further investigation is needed (Innes et al., 2020). Other non-pharmacological avenues that have been studied recently include acupuncture, tension-trauma release exercises, massage therapy, glycerin oil, and lavender oil effleurage massage, largely in populations with an average age of 50–60 years, with varying results that warrant further study in older adults (Kwon et al., 2019; Mirbagher Ajorpaz et al., 2020; Park et al., 2020). Another recent study highlighted significant improvement in the severity of insomnia after cognitive behavioral therapy for insomnia (CBTI) compared to a non-CBTI group in people with a mean age of 55–57, suggesting that this may be another safe, useful treatment modality (Song et al., 2020).

As there are currently no specific guidelines for the management of RLS in the elderly, in utilizing the recommended medications for RLS, careful consideration of potential augmentation and possible side effects in this vulnerable population is important in choosing a management strategy (Table 2). Side effects may include sedation, dizziness, respiratory depression, impulse control disorders, other cognitive disturbances, depression, gait instability, or weight gain (Garcia-Borreguero et al., 2019; Zhang et al., 2019). Side effects may be more common in the older populations (By the 2019 American Geriatrics Society Beers Criteria^®^ Update Expert Panel, 2019), and slow titration up to the lowest effective dose is recommended. Aside from traditional oral therapies, botulinum toxin type A has limited evidence but has shown positive results in the treatment of RLS symptoms, while cannabinoid therapies have not yielded enough evidence to support their clinical use at this time (By the 2019 American Geriatrics Society Beers Criteria^®^ Update Expert Panel, 2019; Su et al., 2021). Further research is warranted for both, as preliminary results have been promising.

**Table 2:** Pharmacotherapy for restless leg syndrome in the elderly (age >65 years) (Garcia-Borreguero et al., 2016; Silber et al., 2021).

**Medication**	**Class of drug**	**Starting dose**	Target dose (Garcia-Borreguero et al., 2016)^*^	Maximum dose (Silber et al., 2021)^*^	**Additional considerations**
Gabapentin Encarbil	Alpha-2-ligands	300 mg	600–1,200 mg	1,200 mg	- Caution with dose escalation in the elderly - Maximum dose listed is not specific to this population. Higher incidence of adverse events are reported (By the 2019 American Geriatrics Society Beers Criteria^®^ Update Expert Panel, 2019). Slow, gradual up-titration is recommended when needed using lowest effective dose - Useful with comorbid insomnia or pain - Side effects: sedation, dizziness, weight gain, constipation - Precautions: use with caution in renal disease
Pregabalin	Alpha-2-ligands	50 mg	150–450 mg	450 mg	- Caution with dose escalation in the elderly - Maximum dose listed is not specific to this population. Higher incidence of adverse events are reported (By the 2019 American Geriatrics Society Beers Criteria^®^ Update Expert Panel, 2019). Slow, gradual up-titration is recommended when needed using lowest effective dose - Useful with comorbid insomnia or pain - Side effects: sedation, dizziness, weight gain, constipation - Precautions: use with caution in renal disease
Gabapentin	Alpha-2-ligands	100 mg	1,200–1,800 mg (Silber et al., 2021) or 900–2,400 (Garcia-Borreguero et al., 2016)	3,600	- Caution with dose escalation in the elderly - Maximum dose listed is not specific to this population. Higher incidence of adverse events are reported (By the 2019 American Geriatrics Society Beers Criteria^®^ Update Expert Panel, 2019). Slow, gradual up-titration is recommended when needed using lowest effective dose - Useful with comorbid insomnia or pain - Side effects: sedation, dizziness, weight gain, constipation - Precautions: use with caution in renal disease
Pramipexole or pramipexole extended release	Dopamine agonist	0.125 mg		0.5–0.75 mg	- Use lowest effective dose, and slow up titration. max dose is 0.75 mg - Increase dose by 0.125 mg every couple of days until symptomatic relief - Useful in severe RLS - Extended-release formulation may have lower risk of augmentation - Side effects: augmentation, leg edema, sedation, impulse control disorders, psychosis - Dose reduction can cause withdrawal
Rotigitine	Dopamine agonist	1 mg		3 mg	- Use the lowest effective dose. Max dose 3 mg - Transdermal patch dosed every 24 h - Useful in severe RLS - Side effects: augmentation, leg edema, sedation, impulse control disorders, psychosis - Dose reduction can cause withdrawal
Ropinirole or ropinirole extended release	Dopamine agonist	0.25 mg	2 mg	4 mg	- Use lowest effective dose. Max dose 4 mg - Most patients 2 mg or less for symptomatic improvement - Extended-release formulation may have lower risk of augmentation - Useful in severe RLS - Side effects: augmentation, leg edema, sedation, impulse control disorders, psychosis - Dose reduction can cause withdrawal

^*^Target and maximum dosage mentioned not specific to the elderly patient; hence caution is advised when increasing the dose from the recommended initial dose.

Additional key considerations for those with RLS in older patients are the increased risk of cardiovascular disease (CVD) associated with RLS. Mounting evidence suggests an association of RLS with cardiovascular risk, showing significantly higher rates of hypertension, diabetes, hyperlipidemia, and other cardiovascular adverse events in RLS patients when compared to controls (Winkelman et al., 2008; Schlesinger et al., 2009; De Vito et al., 2014; Katsanos et al., 2018; Suraev et al., 2020). While there is conflicting data about the relationship between RLS and hypertension, a recent multicenter prospective study in a community-based population with an average age of 65 years demonstrated a significantly increased risk of hypertension in patients with RLS as compared to those without and persisted females in a sex subgroup analysis (Duarte et al., 2020).

Several studies have pointed to a decrease in the physiologic nighttime dip in blood pressure, a known risk factor for CVD, to be linked to RLS symptom severity (Ulu et al., 2015; Guo et al., 2022). One theory behind the mechanism of the blunted blood pressure dip is the fragmented sleep pattern, particularly with concomitant PLMS. Other mechanisms that may play a role in mediating the relationship between RLS and 24-h blood pressure dysregulation include iron deregulation, insulin resistance, sympathovagal imbalance, higher catecholamine activity, genetic factors, and endothelial dysfunction (Ulu et al., 2015). A recent study in Japan demonstrated that RLS in patients with an average age of 65 and heart failure portended lower ventricular function and a worse overall prognosis (Chenini et al., 2019). Another, large, longitudinal cohort study of patients with an average age of 49 at baseline who were free from CVD at the baseline assessment who were followed for an average of 3.4 years, revealed a similar trend, with untreated RLS being associated with a heightened risk of developing CVD after adjusting for confounding variables. Interestingly, the risk of future CVD was significantly lower in those with treated RLS (Yoshihisa et al., 2019). A recent double-blind placebo-controlled trial demonstrated that patients with an average age of 59 and moderate to severe RLS who were treated with rotigotine benefited from higher rates of nighttime blood pressure dips (Gao et al., 2021). Taken together, treating RLS may improve nighttime blood pressure regulation and reduce the risk of CVD.

Another consideration for elderly patients with RLS is the potential association with cognitive decline. There is conflicting evidence of this association, and which cognitive domains may be implicated. Several studies have demonstrated an association between RLS and frontal-executive dysfunction, reduced cognitive flexibility, and reduced attention, while others have shown no association between RLS and cognition (Gao et al., 2021). However, a metanalysis demonstrated a negative association between RLS severity and global cognitive function, as measured by the Mini-mental status exam or MoCA (Chenini et al., 2020), and in another study of patients with an average age of 56, attention was agitated, as measured by the Stroop test or Trails making B test (Jung, 2015). Furthermore, a recent retrospective cohort study of over 2,500 people over 60 years old with up to 12 years of follow-up showed a 1.74 times increased risk of all-cause dementia in people with RLS and a higher risk of developing vascular dementia over Alzheimer's dementia (Xu et al., 2022). The latter finding may be related to the increased risk of CVD that has been suggested in RLS. Moreover, while the cognitive changes that can be seen in people with RLS may be related in part to sleep deprivation, increasing age, or co-morbid depression, it is not clear whether the pathophysiological process of RLS itself impacts cognition directly (Jung, 2015; Colzato et al., 2021).

Conversely, the influence of cognitive decline on the diagnosis and management of RLS should also be addressed. As the incidence of dementia increases with age, it is important to consider the implications of RLS in the subset of older adults with dementia, which can be particularly challenging to diagnose due to limitations in garnering a reliable history in this group. Patients with advanced dementia may experience nighttime agitation that includes aggressive behavior and wandering. A recent study by Richards et al. explored the possibility of nighttime agitation in patients with Alzheimer's disease with a mean age of 73 years as a possible manifestation of RLS (Wang et al., 2023). They studied 76 older adults with a diagnosis of AD and RLS with two nights of observed nighttime behaviors with trained research assistants who assessed RLS using a validated Behavioral Indicator Test-Restless Leg (BIT-RL) and measured agitation using the Cohen Mansfield Agitation Index. In addition, sleep actigraphy on a wrist-worn device and serum iron studies were measured. The investigators found a negative association between transferrin saturation and nighttime agitation suggesting that the BIT-RL may be a useful behavioral measure for assessing RLS in this age group (Wang et al., 2023). In treating RLS in those with cognitive decline, caution should be used with dopamine agonists that can trigger neuropsychiatric consequences of impulse control disorders or psychosis. Studies of the effect of gabapentin enacarbil on possible RLS in patients with dementia and nighttime agitation are currently underway (Richards et al., 2021). More research of the assessment and management of RLS in patients with dementia are needed.

### 3.3 Periodic limb movement disorder

Periodic limb movements of sleep (PLMS) are characterized as stereotyped movements that occur at regular intervals, typically involving the lower extremities of the leg and often presenting with extension of the great toe, the dorsiflexion of the ankle and occasionally flexion at the knee and hip as well (American Academy of Sleep Medicine, 2014). The prevalence of PLMS in people over the age of 60 has been noted to range between 29 and 85% (American Academy of Sleep Medicine, 2014; Kim et al., 2023). PLMS can be seen in three scenarios: first, they may be present in healthy older adults who are often unaware of having PLMS as these movements may not disturb their sleep or functioning (Bliwise, 2006; American Academy of Sleep Medicine, 2014). Secondly, PLMS can be seen in conjunction with other sleep disorders, affecting up to 80%−90% of people with RLS and 70% of people with REM Behavior disorder (Bliwise, 2006; American Academy of Sleep Medicine, 2014). Finally, PLMS can occur as an isolated, frequent movement, affecting the sleep or daytime functioning of the individual, independent of other sleep disorders. The ICSD designates this latter scenario as periodic limb movement disorder (PLMD), which is defined in adults as polysomnographic (PSG) evidence of 15 PLMS/hour with associated sleep disturbance or altered mental, emotional, or physical function that is not attributable to another concurrent sleep disorder (American Academy of Sleep Medicine, 2014).

The pathogenesis and clinical significance of isolated PLMS/PLMD is unclear. Several single nucleotide polymorphisms have been identified that confer greater risk for PLMS and are also associated with RLS, leading to theories that PLMS/PLMD may be a subclinical phenotype on a disease continuum with RLS (Iranzo, 2022). However, further longitudinal studies following people with PLMS/PLMD are needed. Isolated PLMS/PLMD has also been linked to other health conditions. In the Swiss HypnoLaus study, a population-based study of over 2,000 adults with an average age of 58.4 years, participants with a periodic limb movement index (PLMI; number of periodic limb movements per hour) of more than 15/hour had associated changes in sleep architecture with more stage N2 sleep, less N3 and REM sleep and a higher arousal index. These participants also had a higher rate of hypertension, diabetes, and metabolic syndrome (Winkelman et al., 2015). These associations may vary in different ethnic groups, suggesting a more complex relationship between PLMS and CVD risk (Koo and Sillau, 2015; Haba-Rubio et al., 2018). Other studies have suggested a higher prevalence of coronary artery disease and cerebrovascular accidents in patients with higher PLMI (Lin et al., 2018; Shin et al., 2018).

Effective treatment of PLMS associated with RLS parallels the treatment of RLS itself, with lifestyle modifications and pharmacological interventions with alpha-2delta ligands and dopaminergics being the mainstay of treatment (Huang et al., 2019). Few, if any, studies examine the treatment of isolated PLMD that is not associated with RLS. This is an area of unmet need and warrants further exploration, particularly given the potential risk of other health co-morbidities in people with PLMD.

### 3.4 Recent updates in the management of older adults with PLMS/PLMD

As PLMS/PLMD is a common SRMD in older adults, exploring the health implications that PLMS/PLMD may have in this age group is key (Table 1). As mentioned, there is conflicting evidence about the link between PLMS/PLMD and a higher risk of CVD. Since PLMS that is co-morbid with RLS is associated with higher nighttime BP, PLMS is theorized to be associated with higher CVD risk as well (Aurora et al., 2012). The HypnoLaus study demonstrated increased PLMI with age and showed that a PLMI of >15/hour correlated with higher rates of hypertension (Winkelman et al., 2015), but this has not been consistently demonstrated across all ethnic groups (Koo and Sillau, 2015; Haba-Rubio et al., 2018). Del Brutto et al. explored the relationship between PLMS and cerebral small vessel disease in 146 older Ecuadoran adults aged ≥60 years without a prior history of stroke or RLS, evaluating them with PSG and brain MRI. They found no correlation between higher PLMI and imaging markers of cerebral small vessel ischemic disease (Pennestri et al., 2007). To date, there is no clear evidence of a prior history of cerebrovascular disease forecasting a higher risk of PLMS/PLMD (Manconi et al., 2018; Del Brutto et al., 2020).

Aside from potential CVD risk, another consideration for PLMS/PLMD in the elderly is the possible link with cognitive complaints. Studies have suggested a relationship between PLMS and cognitive decline, particularly in those over the age of 60 with Parkinson's disease (Schipper et al., 2018), but this association in the general population remains controversial. In a study of over 2,000 men with an average age of 76 without dementia who were assessed with in-home polysomnography and cognitive assessments, a higher PLMI was associated with a greater decline on Trails Making test- Part B, suggestive of executive dysfunction (Scullin et al., 2015). Another recent study explored the relationship between the PLMI and brain tissue volumes on MRI in 189 adults with an average age of 56 years. Investigators found a significant inverse relationship between high PLMI and reduced volume in key limbic structures, including left and right hippocampus and left amygdala, but clinical correlation with cognitive assessments were not done in this experiment (Leng et al., 2016). In a study by Marchi et al., the investigators did a cross-sectional analysis of 579 community-dwelling individuals with an average age of 71 using PSG studies and a battery of cognitive assessments. They found no association with PLMI, periodic limb movement arousal index (PLMAI), and the cognitive functioning (Szentkirályi et al., 2023). Moreover, with somewhat sparse, conflicting data regarding the potential association of PLMS/ PLMD with cognitive changes, future longitudinal studies that include PSG, clinical data, and imaging biomarkers are needed to further elucidate this relationship.

### 3.5 Nocturnal muscle cramps

Another common sleep-related movement disorder that is common in the elderly is nocturnal cramps. Nocturnal muscle cramps (NMC) are painful, sudden, and intense involuntary muscle contractions of the legs or feet that usually occur during sleep but may occur in wakefulness as well (Monderer et al., 2010; Rabbitt et al., 2016; Marchi et al., 2023). Although they typically last minutes, the discomfort and tenderness from NMC can persist for hours (ISCD-3) (Monderer et al., 2010; American Academy of Sleep Medicine, 2014; Marchi et al., 2023). NMC have been reported to occur in 6%−50% of adults ≥50 years and are more common in women. Although they are typically benign and transient, they can be distressing when it occurs with frequency, affecting one's health-related quality of life and sleep quality (Butler et al., 2002; Grandner and Winkelman, 2017; Sebo et al., 2022).

Abnormal spontaneous motor unit firing and shortened muscle tendons with age are among the many theoretical mechanisms underlying NMC. NMC may be a primary, idiopathic phenomenon or may be secondary to other factors. NMC may be caused by medications that are commonly prescribed in the elderly, including thiazide and potassium-sparing diuretics, long-acting beta-2 agonists, statins, nifedipine, cimetidine, and acetylcholinesterase inhibitors (Table 3) (Monderer et al., 2010; Sebo et al., 2019). Lifestyle factors, including alcohol consumption and sedentary lifestyle, may also contribute to the development of NMC (Garrison et al., 2012; Delacour et al., 2020). They can also be associated with common comorbidities such as diabetes, thyroid disease, uremia, hypomagnesemia, hypocalcemia, or hypokalemia (Marchi et al., 2023). The diagnosis of NMC rests on reliable history and the exclusion of common mimickers such as restless leg syndrome, neurogenic claudication, dystonia, nocturnal myoclonus, or simple muscle strain (Monderer et al., 2010; Rabbitt et al., 2016; Marchi et al., 2023).

**Table 3:** Considerations for managing nocturnal muscle cramps in the elderly.

**Nocturnal muscle cramps**	
General considerations	• Review medication list to identify causative or exacerbating medications • Assess serum chemistry, hemoglobin A1c, thyroid panel to evaluate for uremia, hypokalemia, hypocalcemia, hypomagnesemia, thyroid disease, or diabetes (Marchi et al., 2023)
Common medications that exacerbate nocturnal muscle cramps	• Thiazide and potassium-sparing diuretics • Long-acting beta-2 agonists • Statins • Nifedipine • Cimetidine • Acetylcholinesterase inhibitors
Treatment considerations	• Counsel on effects alcohol consumption and sedentary lifestyle on NMC • Counsel on sleep hygiene • Institute pre-sleep stretching protocol • Consider Magnesium sulfate (limited data) • Consider quinine use for refractory cases with caution. Discuss historical use of quinine, emphasizing the potential risk of serious side effects

Management of NMC is centered on first screening for causative medications, addressing related health conditions, and reviewing lab work to address metabolic derangements and fluid/electrolyte abnormalities (Table 3). Symptoms can initially be addressed with a combination of counseling on sleep hygiene and with pre-sleep stretching (Delacour et al., 2018; Hallegraeff and de Greef, 2020). In a recent, small, randomized-control study in frail adults with an average age of 69, gentle seated hamstring stretches and calf stretches that are each held for 20 s and repeated three times prior to sleep demonstrated a significant reduction in the frequency and severity of NMC without adverse effects (Delacour et al., 2018). Pharmacological treatments have historically centered on the use of quinine due to its effects on reduced motor endplate excitability, but studies have shown mixed results. Additionally, the risk of severe hematological, cardiac, ototoxic, visual, and hepatotoxic adverse effects with quinine limit use (Monderer et al., 2010; Hallegraeff et al., 2012; Rabbitt et al., 2016; Marchi et al., 2023). Magnesium sulfate, vitamin E, vitamin B complex, calcium channel blockers (verapamil and diltiazem), and Naftidrofuryl have limited data but may also have modest benefits (Monderer et al., 2010; Hallegraeff et al., 2012; Marchi et al., 2023). In clinical practice, quinine and magnesium are currently the most widely used pharmacotherapies (Katzberg et al., 2010). There is now a randomized control study underway examining the use of compression stockings vs. magnesium supplement vs. placebo in older adults, but the results are not yet published (Lorenzo et al., 2018). Nevertheless, there remains a need for further research on other effective therapies to address this common problem safely and effectively.

### 3.6 Sleep-related bruxism

Sleep-related bruxism (SB) is a common sleep-related movement disorder characterized by involuntary grinding or clenching of teeth during sleep, caused by stereotyped activation of the masticatory muscles. While often asymptomatic, chronic bruxism may lead to issues such as sleep disturbance, jaw pain, and tooth wear. Self-reported symptoms include teeth grinding or clenching, jaw pain or soreness, headaches, and tooth sensitivity (Wilmont et al., 2019; Joensuu et al., 2021). A 2018 study found that individuals with an average age of 30 and bruxism had a four-time worse oral health-related quality of life, significantly worse sleep quality, and increased daytime sleepiness relative to their non-bruxer counterparts (Câmara-Souza et al., 2019).

Although less prevalent with age, bruxism can occur in the elderly and may be underrecognized (Manfredini et al., 2013; Câmara-Souza et al., 2019; Prakash et al., 2022; Benli and Özcan, 2023; Rauch et al., 2023). Common risk factors and co-morbidities for bruxism include obstructive sleep apnea, use of psychoactive substances, neurologic and psychiatric disorders, and gastroesophageal reflux disease (Benli and Özcan, 2023). In one recent study of 191 German seniors ≥60 years old, 16.8% of the study sample self-reported having SB (Prakash et al., 2022).

The exact pathophysiologic mechanism behind SB remains elusive. Studies indicate that SB is influenced by impaired motor control and activation of the sympathetic nervous systems during sleep (Smardz et al., 2021). The diagnosis of sleep-related bruxism is made through clinical interview, examination, corroboration by parents/caregivers or bed partners, and polysomnography if there is uncertainty about whether the events described are attributable to sleep-related bruxism (Câmara-Souza et al., 2019; Joensuu et al., 2021; Smardz et al., 2021). Since occasional bruxism is common and often asymptomatic, many individuals do not require specific treatment (Joensuu et al., 2021). However, frequent, or symptomatic sleep-related bruxers may benefit from oral appliances, behavioral interventions, or pharmacotherapy (Joensuu et al., 2021).

For individuals with frequent bruxism or related tooth wear, the use of a medically fitted oral device that covers the upper and lower teeth remains a cornerstone of therapy for SB. Oral appliances have been shown to protect the teeth from damage and reduce grinding noises associated with bruxism (Joensuu et al., 2021). There are two commonly used categories of oral devices available for use: occlusal (bite) splints and mandibular advancement splints (MAS) (Landry et al., 2006; Alóe, 2009). In a recent study of college students comparing these devices, patients self-reported increased comfort with occlusal splints, but MAS showed a greater reduction in the index of nighttime rhythmic masticatory muscle activity (RMMA) on polysomnography (Landry et al., 2006). Though in a study of patients with an average age of 25.5 years, more affordable, over-the-counter (OTC) fabricated splints have demonstrated less efficacy in reducing RMMA bursts compared to those devices created by dental professionals (Abe et al., 2023). Massage therapy and Kinesio tape may also play a role in treating SB (Keskinruzgar et al., 2019; Gerstner et al., 2020). Biofeedback splints that can provide sensory feedback in response to nocturnal masticatory clenching is another area that is under investigation for the treatment of SB, but concerns regarding the potential disruptive effects on sleep are an important consideration (Ilovar et al., 2014; Volkan-Yazici et al., 2021).

There is increasing evidence for chemodenervation with botulinum to manage refractory SB. Several case series and small randomized trials of younger patients, ranging in age from 18 to 50 years, have previously demonstrated an improvement in the number of bruxism events, pain, or range of masticatory motion with the injection of the masseter muscles and/or temporalis muscles with botulinum toxin (Table 4) (Long et al., 2012; Bergmann et al., 2020; Kaya and Ataoglu, 2021). While there is limited data on bruxism in older adults, case reports suggest that bruxism may be a concern to vulnerable geriatric populations such as those with dementia and that botulinum injections may be an appropriate management strategy (Ondo et al., 2018). Larger studies are necessary to confirm the efficacy of this treatment, but current evidence suggests that BoNT-A injections may be an effective option for the management of SB, especially for individuals who are refractory to other treatment options.

**Table 4:** Considerations for managing sleep-related bruxism in the elderly.

**Sleep-related bruxism**	
General considerations	• May be underrecognized in the elderly • Treatment with oral appliances is first line therapy • Botulinum toxin may be beneficial and may be preferred in cognitively-impaired individuals. • Consider kinesio-tape and masseter muscle massage as alternative therapies
Botulinum toxin therapy recommendations (limited data)	• Masseter: 24–30 units of Botox (two sites) (Long et al., 2012; Bergmann et al., 2020; Kaya and Ataoglu, 2021) or 80 units of Dysport (Bergmann et al., 2020) • Temporalis: 20 units of Botox (three sites) (Bergmann et al., 2020; Kaya and Ataoglu, 2021)

## 4 Conclusion

Sleep related movement disorders are common in individuals over age 55, and may be underrecognized by patients and providers (Mathew et al., 2020). Routine screening assessments of sleep health at primary care visits, even with the simple questions on the commonly used Patient Health Questionnaire-9 (PHQ-9), may alert a provider to symptoms of poor sleep and prompt more detailed sleep history-taking (Senthilvel et al., 2011; Gordon et al., 2022; Yamamoto et al., 2023). Collateral history from a partner or caregiver can provide additional insight into a person's nighttime behaviors and movements, triggering further work up for SRMD like RLS, PLMD, NMC, or SB as discussed in this review.

The current management recommendations for SRMD are nearly uniform across all ages. However, a more personalized approach, with attention to factors that are prominent in older adults should be considered. For example, certain factors, including the potential for medication-induced SRMD in the setting of polypharmacy, the increased risk of adverse side effects to pharmacotherapy in older adults, and the challenges of recognizing of SRMD in vulnerable sub-populations such as those with dementia, may be unique considerations in this population. Furthermore, additional research on SRMD in the elderly, such as examining the possible association of restless leg syndrome with the risk of nighttime falls in the elderly, is also warranted to explore the downstream impact of these disorders. Healthy sleep is known to promote healthy aging (Miner and Kryger, 2017; Gulia and Kumar, 2018). Given the prevalence of SRMD in the elderly, clinical attention and future research focusing on the safe and effective management of SRMD in this population is needed.

## Author contributions

MC: Data curation, Formal analysis, Investigation, Resources, Writing—original draft, Writing—review & editing. SA: Data Curation, Formal analysis, Investigation, Writing—original draft, Writing—review & editing. PG: Conceptualization, Data curation, Formal analysis, Investigation, Methodology, Project administration, Supervision, Writing—original draft, Writing—review & editing.
